# Costing the long-term health harms of trafficking: Why a gender-neutral approach discounts the future of women

**DOI:** 10.3389/fsoc.2022.858337

**Published:** 2022-09-08

**Authors:** Sylvia Walby, Estela Capelas Barbosa, Sally McManus

**Affiliations:** ^1^Violence and Society Centre, City, University of London, London, United Kingdom; ^2^National Centre for Social Research, London, United Kingdom

**Keywords:** trafficking in human beings, economic cost analysis, health harms, gender disaggregation, sexual exploitation, labor exploitation

## Introduction

When the European Commission published a major report estimating the costs - health, social, and economic - of the trafficking in human beings in Europe (Walby et al., 2020) the decision was made to not apply the common economic practice of “discounting”. Discounting involves reducing the estimated value of the future relative to the present, year by year, by a given percentage. As authors of that report, we instead opted to more fully recognize and account for the longer-term costs to mental health. We argue that a methodological approach that does not systematically discount the future can bring into focus the greater long-term health burden faced by women who have been trafficked, and in particular by the victims of trafficking for sexual exploitation.

## Estimating the costs of trafficking in human beings

The mental health harms of human trafficking have long been recognized (Beyrer, 2004). Health is affected during trafficking (Ottisova et al., 2016), but the consequences of trauma can be long-lasting (Kiss et al., 2015; Oram et al., 2015). While injuries sustained during the trafficked period are generally included in economic costings, UK government costings tend to not account for the long-term health consequences, as well as take a “gender neutral” approach (Reed et al., 2018).

While trafficking in human beings does not need to be costed in order to affirm that it is immoral, a serious crime, and a breach of human rights, the practice of costing social ills can help legitimate the use of public funds to address them (Walby and Olive, 2014). If something costs society a certain amount, then it could be cost effective to spend that amount preventing it. While most agree that this cost to society includes the use of public services (law enforcement, health care, and specialized victim-support services), lost economic output, and a value placed on the lost quality of life, other specific aspects are contested and less well evidenced, such as the number of years for which individuals are affected. Existing data collection and research has faced methodological limitations, such as small or unrepresentative samples, cross-sectional designs, and a lack of information on gender.

## Including the longer-term harms to health

In 2018, the UK government published an estimate of the cost of “modern slavery” in the UK (Reed et al., 2018). It counted use of services, lost economic output, and “intangible” costs accrued during the trafficked period, plus some costs during a recovery period of up to five years. Any longer-term harms (such as those related to ongoing anxiety and depressive disorders and health service use) were not included in their estimates, in part due to uncertainty about the duration of effects. The estimates produced for the UK using their approach found little per victim difference in the cost to society between those trafficked for labor exploitation and those trafficked for sexual exploitation. While they provided no gender-disaggregated estimates at all, the implication was that male and female victims of trafficking experienced similar levels of harm despite female victims being more exposed to sexual violence (Rose et al., 2020).

In contrast, our costing for the European Commission of the costs of trafficking of human beings in Europe included longer-term harms to mental health. This enabled us to better distinguish costs associated with trafficking for sexual exploitation, which in our work were far higher (€364,474 per victim) than the costs associated with trafficking for labor exploitation (€232,923 per victim). Since women are more likely to be trafficked than men, as well as more likely than men to be trafficked for sexual exploitation, this approach found that the total costs of trafficking in Europe associated with female victims (€2,168,738,120) were almost four times greater than those for male (€540,052,406).

## Challenging the economic practice of “discounting” long-term outcomes

Long-term health matters to costings of other forms of violence and abuse, including domestic violence and child sexual exploitation. By not including the long-term consequences for mental and physical health, health service related costs are under-recognized by the UK government's policy makers and Treasury, as are the greater health harms specifically related to sexual violence (Walby et al., 2017). Further, discounting - a practice through which the present is systematically valued more than the future - reduces the “value” of the future, year by year, by a given percentage. While this is a routine practice in economics and features in the estimates generated for many governments around the world, the health-focused Global Burden of Disease project (alongside many environmentalists) soundly rejects it. They argue that: “discounting should not be applied to future health gains or losses because health is not commensurable with money and cannot be reinvested elsewhere… Discounting counted years of healthy life saved in the present as more valuable than years of life saved in the future.”(Institute for Health Metrics Evaluation, 2013). Economist Nicolas Stern has argued that there is no serious ethical argument in favor of pure-time discounting; that it is essentially “discrimination by date of birth” (Stern, 2018).

## Discussion

Our estimates of the costs of human trafficking did not apply the economic practice of discounting, preferring to leave decisions as to the relative value of the future and the present to policy makers. The current “gender neutral” approach in many government costings fails women, fails to capture gender variation, and fails future generations. We ask economists to review practice and include fuller recognition of the longer-term harms to health of violence in their cost-benefit analyses and to re-examine the practice of discounting long-term health costs. This should lead to a re-evaluation of the public funds to support the health needs of these most exploited of human beings.

## Author contributions

All authors contributed to the conceptualization, drafting, and editing of this article. All authors contributed to the article and approved the submitted version.

## Funding

The writing of this article was supported by the UK Prevention Research Partnership (Violence, Health and Society; MR-VO49879/1), which is funded by the British Heart Foundation, Chief Scientist Office of the Scottish Government Health and Social Care Directorates, Engineering and Physical Sciences Research Council, Economic and Social Research Council, Health and Social Care Research and Development Division (Welsh Government), Medical Research Council, National Institute for Health Research, Natural Environment Research Council, Public Health Agency (Northern Ireland), The Health Foundation, and Wellcome. The views expressed in this article are those of the authors and not necessarily those of the UK Prevention Research Partnership, Commission, or any other funder.

## Conflict of interest

The authors declare that the research was conducted in the absence of any commercial or financial relationships that could be construed as a potential conflict of interest.

## Publisher's note

All claims expressed in this article are solely those of the authors and do not necessarily represent those of their affiliated organizations, or those of the publisher, the editors and the reviewers. Any product that may be evaluated in this article, or claim that may be made by its manufacturer, is not guaranteed or endorsed by the publisher.
